# Breast cancer knowledge and awareness among university students in Angola

**Published:** 2012-04-16

**Authors:** Martha Nyanungo Sambanje, Benford Mafuvadze

**Affiliations:** 1 Faculdade de Ciências da Saúde, Universidade Metodista de Angola; 2 Department of Biomedical Sciences, University of Missouri, Columbia, MO 65211, United States of America

**Keywords:** Breast cancer, knowledge, university students, self-examination, Angola

## Abstract

**Background:**

The high breast cancer mortality rate in Sub-Saharan Africa has been attributed to a lack of public awareness of the disease which often leads to late diagnosis of the disease. Little is known about the level of knowledge and awareness of breast cancer in Angola. Previous studies have shown that breast cancer awareness is higher among well-educated people. The goal of this study was to assess breast cancer knowledge and awareness among university students in Angola.

**Methods:**

We conducted a cross-sectional survey of university students using a self-administered questionnaire to investigate participants’ awareness and knowledge of breast cancer. A total of 595 university students in medical and non-medical programs successfully completed the survey.

**Results:**

Our results showed insufficient knowledge of breast cancer among university students in Angola irrespective of whether they were in medical or non-medical programs. The majority of the participants were not aware of some of the early signs of breast cancer such as change in color or shape of the nipple, even though they appreciated the need for monthly breast self-examination. Overall most of the participants indicated the need for increased breast cancer awareness among university students.

**Conclusion:**

The study points to the insufficient knowledge of university students in Angola about breast cancer. We expect that our results may provide useful data that may be used by the department of health in Angola and other African countries to formulate health education programs aimed at increasing awareness and promote screening and early detection of breast cancer in the continent.

## Background

Breast cancer is the most common type of cancer in women worldwide. There has been a significant increase in the incidence of breast carcinoma in sub-Saharan African countries and in other low-resource countries [1, 2]. In comparison to western countries, breast cancer in African women tends to occur in premenopausal women with incidence peaking between the ages of 35 and 45 years [3, 4]. Similar to African-American women in the US, breast cancer in African women tend to be the aggressive triple negative subtype [5, 6], which is non-responsive to commonly used therapeutic drugs. Mortality rates for African women are relatively higher when compared to women in Western countries [1, 7]. Unless medical care and screening practices are dramatically improved in Africa, breast cancer mortality rates can be expected to remain disproportionately high [8]. Apart from African women being predisposed to the more aggressive form of breast cancer, the disproportionately greater mortality rate compared to high-resource countries can be attributed to a lack of public awareness of the disease, absence of organized screening programs, delayed presentation and lack of accessible and effective treatment options [9]. As a result of late detection, most patients are diagnosed well after the breast cancer is at an advanced stage and has metastasized to other organs [4]. For example, previous studies have reported that 70-90% of African women present with stage III or IV breast cancer [10, 11]. The advanced stage distribution is partially explained by delayed presentation for medical evaluation, which, according to Anyanwu [12], can be as high as 11 years from the time of self-detected breast abnormality.

While in Western countries, breast cancer screening is usually done using mammograms, the use of mammograms is limited and inaccessible to most women in sub-Saharan Africa. This situation is unlikely to change in the foreseeable future [13]. In the absence of readily available mammographic screening, despite its known limitations, breast self-examination (BSE) remains a viable and practical alternative for African women [13, 14]. With greater awareness of breast cancer and proper training in BSE combined with regular clinical breast examination, it is possible to diagnose breast cancer earlier. Women who regularly perform BSE become familiar with both the appearance and feel of their breasts which often helps them detect any changes early. However, if improperly done, BSE has the risk of giving false health security and may actually reduce willingness to undergo mammographic screening even in place where it is readily available [15].

Awareness and understanding of breast cancer in Africa is generally low. In recent years, the World Health Organization and several international organizations such as The Breast Health Global Initiative (BHGI) have sought to increase breast cancer awareness among African women [16, 17]. Studies in other parts of the world have shown that general breast cancer awareness increases with level of education [18]. Little is known about the knowledge level and awareness of breast cancer among university students in Angola. The main purpose of this study was, therefore, to assess breast cancer knowledge and awareness among university students in Angola. Because breast cancer affects African women early as previously mentioned, most university students are at a stage where it is critical that they at least perform BSE regularly and potentially detect any changes early. Previously, most studies aimed at investigating the level of understanding of breast cancer among college students focused on female students [19–22]. We chose to include male students in our study because previous studies have shown widespread lack of knowledge of the disease among men [23]. It is our argument that even though breast cancer is not common in men, well-informed men can play a significant role in increasing awareness among the general public.

## Methods

This study was designed to evaluate the level of breast cancer awareness among university students in Angola. In particular, we focused on perceptions about the causes of breast cancer, risk factors associated with breast cancer and knowledge of breast self-examination. Participants were drawn from students enrolled in Medical and Health related programs and non-medical programs from six universities in the capital Luanda. Permission to conduct the study was sought and granted by the University of Metodista de Angola's Review Board. At participating institutions, the purpose of the study was explained to participants and those who freely agreed to participate were enrolled in this study. A total of 595 students (350 females and 245 males) volunteered and successfully completed the survey. Participants in non-medical programs were drawn from a teacher-training, foreign language and biological science programs.

### Instruments

We used a modified version of the Breast Cancer Perceptions and Knowledge Survey previously used by Powe et al [24]. The questionnaire was translated into Portuguese and reviewed for common language use. The instrument was pilot tested on a group of 30 university students that were randomly selected on campus in order to determine if there was an ambiguity in the wording. Results of the pilot test were used to modify the wording of questions in order to maximize their easy understandability before the questionnaire was administered to participants in this study.

We adapted items for use in this study by including the response, “I don't know” in addition to the yes (agree)-no (disagree) format used by Powe et al [24] on the section on general breast cancer knowledge, and in order to fully assess knowledge of a particular risk factor, we asked the participants whether a given factor would, increase, decrease, had no effect on breast cancer risk or alternative indicate if they “don't know”. The questionnaire used in this study had a total of 25 questions, of which 13 assessed knowledge about breast cancer risk factors, 12 assessed perceptions of breast cancer and general knowledge about breast self-examination. Each correct answer (based on American Cancer Society, [25] was assigned a score of 1, while an incorrect answer or “don't know” was awarded a score of 0. A total score for each participant was computed by summing the number of correct answers.

A demographic section of the questionnaire was used to acquire participants’ information such as gender, age, high school attended, marital status, current academic class, and knowledge of someone with breast cancer. Based on previous studies [15], we hypothesized that these factors could potentially influence breast cancer knowledge among the participants. We also asked participants’ opinion on provision of information on breast cancer awareness as part of the curriculum in universities.

### Statistical analysis

Data were analyzed using Statistical Package for the Social Sciences (SPSS, Version 19). Descriptive statistics with cross-tabulations were performed and frequencies were generated for correct and incorrect answers for all items. As previously stated, the knowledge indices were calculated for each student by summing the number of correct answers. Participants were grouped depending on whether they were in a medical program or non-medical program. Pearson Chi-Square test was used to examine the association between variables, with significance level set at p

## Results

Table 1 describes the socio-demographic nature of the respondents; the majority of the students were aged between 21 and 25 (51% of Medical students and 57% of non-medical students) and most were single. Most of the medical students (83.5%) in this study were still in the preclinical stage of their studies and therefore had not been exposed to mandatory curriculum knowledge on breast cancer. The majority of the participants (nearly 60%) had attended public schools for their high school education. Our results also showed that less than 20% of the participants reported knowledge of someone with breast cancer.

**Table 1:** Demographic characteristics of participants

	Medical students (N=320)	Non-medical students (N=275)
Characteristic	% (n)	% (n)
**Gender**		
Male	39 (126)	43 (119)
Female	61 (194)	57 (156)
**Age (Years)**		
<20	30 (95)	35 (97)
21-25	51 (162)	57 (156)
26-30	13 (42)	5 (13)
>30	7 (21)	3 (9)
**Marital status**		
Single	94 (302)	95 (261)
Married	4 (14)	4 (11)
Divorced	1(4)	1(3)
**High school attended**		
Public school	58 (187)	60 (165)
Private school	25 (81)	30 (81)
Mission school	7 (23)	6 (16)
Other	4 (13)	5 (13)
**Academic Year**		
First year	45(144)	24 (67)
Second year	25 (79)	43(119)
Third year	14 (44)	12 (32)
Forth or more	17(53)	21 (57)
**Knowledge of someone with breast cancer**		
Yes	18 (59)	19 (53)
No	82 (261)	80 (219)

*Values may not add up to 100% due to missing values or rounding up to 1 decimal place

### Breast cancer knowledge

This study assessed breast cancer knowledge and awareness among university students in Angola (Table 2). We particularly focused on students pursuing non-medical related programs and those in medical programs but still in preclinical stages as we wanted to remove the bias brought by esoteric knowledge acquired in mandatory medical curriculum. Our results showed no significant difference (p>0.05) between students in medical programs and those in non-medical programs as both generally showed lack of adequate knowledge on breast cancer. Our results also showed that most of the students (nearly 65%) were not aware of the fact that breast cancer is one of the most prevalent cancers in women in Angola. Similarly, less than 35% of the students were aware of the fact that breast cancer can affect men. The majority of participants (more than 80%) subscribed to the misperception that lumps in breasts that are cancerous are painful. It was also clear that most of the participants were not aware of noticeable indicator that can point to breast cancer. For example, less than 40% of the participants were aware of the fact that changes in color or shape of the nipple could be a sign of breast cancer. Thus, even though a significant number of the participants (more than 80%) indicated that monthly BSE might help in early detection of breast cancer; most of them were not knowledgeable on how to perform it. This was further confirmed by the fact that nearly half of the participants were not aware of the best time of their menstrual cycle to perform BSE. It was, however, interesting to note that participants did not subscribe to general misperceptions such as breast cancer is common in women with big breasts, being hit on the breast can cause breast cancer or a woman who lets a man put “love bites” on her breast is more likely to get breast cancer. Furthermore, a majority of the participants (66%) were aware of the fact that if breast cancer is diagnosed and treated early, there is a higher chance of survival.

**Table 2:** Perceptions and knowledge of breast cancer

Question	Medical students (N=320)			Non-Medical students (N=275)		
	Cor	Incor	D/K	Cor	Incor	D/K
Breast cancer is one of the most prevalent cancers in women in Angola	37 (118)	46 (148)	15.6 (50)	34.5 (95)	42 (115)	20 (56)
Breast cancer can affect men	30 (97)	39 (126)	26 (83)	33 (91)	43 (119)	20 (55)
Women younger than 30 years do not get breast cancer	65 (207)	12 (38)	18 (59)	71 (195)	9 (24)	18 (48)
Breast cancer is more common in women with big breasts	70 (224)	5 (17)	21 (68)	67(185)	6 (17)	22 (61)
Lumps in the breast that are cancer are usually painful	14 (46)	55 (176)	28 (88)	20(55)	50 (137)	25 (69)
Being hit on the breast can cause cancer	52 (165)	12 (38)	33 (105)	50 (138)	14 (39)	32 (87)
A woman who lets a man put “love bites” on her breast is more likely to get breast cancer	75 (241)	5 (16)	18 (57)	66 (181)	10 (28 )	25 (59)
One of the best ways to find breast cancer early is by checking the breasts every month (breast self-examination)	84 (270)	6 (19)	8 (27)	78 (215)	8 (23)	10 (28)
The best time to check for lumps in the breast is just after the period ends	47 (149)	8 (27)	43 (138)	47 (130)	11 (31)	38 (105)
A change in the color or shape of a woman's nipple could be a sign of breast cancer	38 (120)	20 (64)	41 (130)	36 (98)	18 (49)	45 (117)
A woman's chance of surviving breast cancer is very low, even if it is found early	66 (210)	14 (46)	19 (60)	66 (180)	14 (39)	18 (48)
Black African women are more likely to develop breast cancer than white women	78(236)	4 (11)	22 (71)	55 (151)	8 (22)	35 (96)

Note Cor=Correct, Incr= Incorrect, D/K= Don't Know;

*Values might not add up to 100% due to missing values and rounding off to 1 decimal place.

### Understanding of risk factors

Our results showed a widespread lack of knowledge on risk factors associated with breast cancer. Participants who reported knowledge of someone with breast cancer were slightly more knowledge than those who did not. All the other variables such as age, gender, academic level, high school attended and marital status did not have a significant influence on level of understanding of risk factors. Additionally, no differences (p<0.05) were observed between students in non-medical programs and those in medical programs. As shown in Table 3, the most known risk factor was a family history of breast cancer. On the other hand, participants were generally not aware of the long term risk posed by oral contraceptives, hormone replacement therapy and obesity or the potential benefits of breast feeding and early child birth (Table 3).

**Table 3:** Understanding of breast cancer risk factors

Risk factor	Medical students (N=320)			Non-med students (N=275)		
	Cor % (n)	Incor %(n)	D/ K %(n)	Cor % (n)	Incor % (n)	DK %(n)
A family history of breast cancer	46(148)	28 (88)	20 (64)	55 (150)	18 (50)	26(70)
Hormone replacement therapy (HRT)	13 (43)	23 (69)	56 (178)	11 (31)	23 (62)	63 (173)
Eating fatty foods, with little vegetables	46 (146)	10 (33)	38 (121)	39 (108)	10 (27)	49 (134)
A stressful life	5 (16)	65 (209)	23 (75)	5 (14)	61 (168)	32 (89)
Regular exercise	1 (4)	59 (189)	31 (100)	5 (13)	55 (152)	37 (102)
Having children before 30 years of age	60 (193)	16 (52)	16 (52)	58 (160)	17 (46)	24 (65)
Wearing tight bras	30 (95)	29 (94)	34 (109)	28 (78)	27 (74)	42 (116)
Being overweight	38 (121)	22 (69)	35 (111)	35 (87)	33 (90)	34 (93)
Using oral contraceptives	38 (120)	15(47)	43 (136)	25 (68)	11 (30)	62 (170)
Mammograms	13 (40)	33 (106)	47 (151)	15 (41)	34 (93)	49 (135)
A hard blow to the breast	11 (34)	39 (124)	44 (141)	14 (38)	34 (94)	47 (128)
Breast feeding	2 (5)	53 (170)	40 (129)	8 (21)	43(117)	48 (131)
Breast Implant	28 (88)	39 (125)	28 (91)	26 (72)	27(73)	44 (122)

Note Cor=Correct, Incr= Incorrect, D/K= Don't Know;

*Values might not add up to 100% due to missing values and rounding off to 1 decimal place

### Understanding of breast self-examination

Our results showed that 69.4% and 72.4% of medical and non-medical students respectively reported having knowledge of BSE. However, it was interesting to note that only 40.2% of female medical students and 38.7% non-medical female students reported that they could confidently perform BSE. Given that a majority of the students (more than 60%) where not aware that a change in the color or shape of the nipple could be a sign of breast cancer and more than 50% did not know the best time to perform BSE (Table 2), it remains to be investigated whether the students who claimed that they are confident to perform BSE indeed could do it properly.

### Overall knowledge

Overall, our study is in agreement with previous studies done in other parts of the world and showed the general lack of adequate knowledge on breast cancer by university students in Angola. The overall mean score for Medical students was 9.9 ± 0.25 (mean ± SEM) and for non-medical students, 9.4 ± 0.21(mean ± SEM) out of a total possible score of 25. The only variable that had a significant effect on breast cancer awareness was knowledge of someone with breast cancer, with participants who knew someone with breast cancer being significantly more knowledgeable (p<0.05) than those who indicated that they did not know anyone with breast cancer. Other variables such as gender, age, marital status, and high school attended did not have a significant influence on level of knowledge. Interestingly, most of the participants (97.5% of medical students and 98.5% of non-medical students) indicated the need for more information on breast cancer to be provided in high school and university, thus, suggesting a willingness to learn more about the disease.

## Discussion

Breast cancer mortality rate is much higher among Sub-Saharan women as compared to women in Western countries, even though the incidence rate is much higher in Western women [1, 2]. Apart from the fact that African women develop a more aggressive form of breast cancer, the higher mortality rate has been attributed to a general lack of public awareness of the disease, coupled with limited screening programs which often results in late diagnosis of the disease even after it has already metastasized to other organs [9]. WHO and several international and local organizations have, in recent years, begun campaigns to increase breast cancer awareness among women throughout Sub-Saharan Africa. Breast cancer occurs much earlier in African women reaching a peak 10 years earlier (35-40 years) making it more important to increase breast cancer awareness at an earlier age. The main focus of this study was to assess breast cancer awareness and knowledge among university students. Our overall results, in agreement with studies done in other parts of the world [19–22, 26–28], showed a general lack of breast cancer awareness and knowledge among university students irrespective of their gender, marital status, years in university and nature of high school attended. Interestingly, perceptions about breast cancer reported by students in medical programs were no different from those in non-medical programs. Previous studies done in different parts of the world have shown that there is have very limited knowledge of breast cancer even among Healthcare professions like nurses [24, 27, 29]. Since we deliberately did not include clinical medical students in this study, it would be interesting to investigate how knowledgeable the same students would be after going through their clinical courses.

In contrast to a study done by Mafuvadze et al [30] who reported 72% of university students reporting knowledge of someone with breast cancer in the USA, in this study, less than 20% of participants reported knowledge of someone with breast cancer. In line with what was reported by Wadler et al [9], this is possibly due to the secretiveness to which having breast cancer is viewed in Africa. Given that students with knowledge of someone with breast cancer were slightly more knowledgeable than those who did not, it is possible that breast cancer survivor can play a significant role in bringing awareness among the general public.

A majority (80%) of participants in this study shared the view that lumps in the breast that are cancerous would be painful. This, as reported by Powe et al [24], is a widespread misconception as most people associate pain with the occurrence of cancer. The fact is that pain in not necessarily an early sign of breast cancer. Ukwenya et al [10] reported in a study in Nigeria that a majority of breast cancer patients cited ignorance of the seriousness of a painless lump as a reason for prolonged delay before seeking medical advice. Thus, without full knowledge, affected women will still take so long to consult medical experts even if they notice some changes in their breasts. Interestingly, even though a majority of the participants appreciated the need for monthly BSE, it is evident that most students have not received proper information on how to properly perform it. In addition, a majority of the participants were not aware of early signs of breast cancer such as changes in color or shape of the nipple, implying that they are likely to miss these even if they perform what they deem is BSE. According to McCready et al [15], improperly conducted BSE can lead to false assurance of health and lead to delayed seeking of medical attention.

The majority of students in this study showed a general lack of understanding of some of the common risk factors associated with breast cancer. Although some of these risk factors are not easily modifiable, others, such as the use of oral contraceptives and hormone replacement therapy (HRT), alcohol consumption, and physical inactivity can be altered in order to reduce risk. In this study, more than 60% of students were not aware of the risk associated with use of oral contraceptive and hormone replacement therapy. University students are at a stage where they are making important reproductive decisions such as use of oral contraceptives. They should, therefore, be aware of the risk associated with long term use of oral contraceptives. Though use of hormone replacement therapy is not common in Africa at present, it is, however, likely that a significant number of women will have access to it as African economies develop. Though a significantly high number (60%) of students were aware of the fact that having children early might reduce the risk of breast cancer, less than 8% were aware of the benefit of long term breastfeeding in reducing breast cancer risk. Studies in other parts of the world reportedly showed that well-educated and higher socio-economic status level women were more likely to formula feed than breast-feed [31–33]. In addition to the other benefits of breast-feeding to the baby, it is important that women be informed of the potential effect on breast cancer risk.

There is not much information on breast cancer awareness among men in Africa. Men tend to view breast cancer as a female only disease as revealed in a study by Thomas [23] where men even voiced concern that a diagnosis of breast cancer would cause them to question their masculinity. We included men in our study because knowledgeable men can be sources of knowledge and can act as pillars of support to affected partners, relatives and friends [34]. In agreement with a previous study by Thomas [23], nearly 70% of participants in our study were not aware of the fact that men can be affected by breast cancer and the risk may be higher with a family history of the disease. Thus, even though breast cancer is less common in men, they still need to be made aware that they can get the disease. We recommend that, where possible, breast cancer awareness programs should also target men.

In Angola, like in many Sub-Saharan countries, there is a general unwillingness to openly discuss issue pertaining to reproductive health [35]. However, more than 97% of the participants in this study indicated a need for increasing breast cancer awareness among university and college students suggesting that there is a general willingness to learn about breast cancer. It is evident from this study that there is need for health educators to come up with culturally acceptable programs to increase general breast cancer awareness among university students.

While a lot of attention in Sub-Saharan Africa has been on HIV and AIDS in the last three decades, it is important to also focus on other important disease like breast and cervical cancers which continue to cause high mortality among women [8]. Health care and education providers can possibly exploit already established platforms to increase public awareness of these other disease as well. In addition, there is also need to be innovative and come up with methods that increase dissemination of information on breast cancer in culturally acceptable ways. Most women in Africa are uncomfortable to discuss issues pertaining to reproductive health with men [35]. As suggested by Galukande and Kiguli-Malwadde [36], in order to foster the transfer of breast cancer knowledge to women, involvement of trained non-physician personnel as well as adoption of non-conventional places as distribution centers of information such as hair salons, banks, grocery shops, *et cetera* may lead to increased awareness. For example, recently, in Zimbabwe programs were begun in which trained hairdressers distribute and disseminate information on reproductive health [37]. In addition, as suggested by Sait et al. [21], breast cancer awareness programs should be developed in universities including lectures, seminars, workshops and on hands training. The concept of National Month of Breast Cancer Awareness should also be embraced in Angola as is the case in most Western countries.

## Conclusion

Our results showed a general lack of knowledge of breast cancer among university students in Angola irrespective of whether they were in medical programs (prior to clinical years) or non-medical programs. We contend that these findings further highlight the need for developing and implementing effective breast cancer education and prevention programs among university and the general public in Angola. We expect that our results may provide useful data that may be used by health institutions in Angola and other African countries to formulate health education programs focusing on breast cancer that targets university students.

We think that the apparent lack of breast cancer awareness and knowledge among university students in this study is further evidence for widespread ignorance among the general public. University students tend to be better informed on health issues than the average public person [35]. It is, therefore, crucial that health care providers and educators employ culturally appropriate strategies to increase breast cancer awareness.

Establishment of trained breast cancer awareness peer groups at universities and other educational institutions in line with those currently promoting HIV awareness would likely increase breast cancer awareness. The last few years have seen widespread use of internet social media such as *Facebook* and *Twitter* in Africa. There is a lot of potential to exploit these online social media as a means of disseminating information on breast cancer.
